# Experimental determination of concentration factors of Ni, Ru and Sb in the model diatom *Phaeodactylum tricornutum*

**DOI:** 10.1038/s41598-023-38795-2

**Published:** 2023-08-21

**Authors:** Y. Ali, R. Thomas, S. Holgersson, M. Isaksson, K. Insulander Björk

**Affiliations:** 1 Department of Medical Radiation Sciences, Institute of Clinical Sciences, Sahlgrenska Academy, University of Gothenburg, Gothenburg, Sweden; 2 Division of Energy and Materials, Department of Chemistry, Chalmers University of Technology, Gothenburg, Sweden

**Keywords:** Ecosystem ecology, Marine biology, Marine chemistry, Nuclear chemistry

## Abstract

This paper describes the experimental determination of concentration factors (CF) for nickel, ruthenium and antimony in the model diatom *Phaeodactylum tricornutum Bohlin (Bacillariophyceae)*, which was chosen as a representative of marine phytoplankton. Better determinations of these CF are needed to improve the modelling of marine ecosystems at release points, where radioactive pollutants enter the ecosystem, for more accurate predictions of radiation dose to humans caused by these pollutants. A literature study revealed that the currently implemented values of these CF are based on very scarce data, and a computational sensitivity study showed that the radiation dose caused by radioisotopes of these elements depend strongly on the phytoplankton CF. Nutrient-enriched water samples from Swedish coastal waters were used as a medium for growing of the diatom species *P. tricornutum* and radioactive isotopes of the studied elements were added to the cultures during the exponential growth phase. The radioactivity in the *P. tricornutum* and in the culture medium were measured separately and used for determination of CF. Conservative estimates of the CF based on this phytoplankton proxy on the present data are 6400 L/kg for nickel, 20,000 L/kg for ruthenium and 890 L/kg for antimony, with *P. tricornutum* biomass masses referring to dry weight. The estimates for nickel and ruthenium are similar to previously published values, which underpins the credibility of radiation dose calculations based on these values. The estimate for antimony is uncertain, but also, to our knowledge, represents the first published experimentally based data on this CF.

## Introduction

Radionuclides are among the various contaminants released into marine ecosystems through discharges linked to human activities, most predominantly from the nuclear industry, both during normal operations and from unforeseen releases such as nuclear reactor incidents and accidents^1, 2^. The assessment of the resulting radiation dose to various recipients requires monitoring of the releases and studies on how the radionuclides are taken up by various organisms within food webs. Since nuclear power reactors are frequently situated on coastal areas, the marine ecosystems are often the recipients of radionuclide releases, and hence the transport of radionuclides within these ecosystems must be studied. In the marine environment, estimates of release and uptake of radionuclides are mostly based on simulation models with limited experimental data (e.g. PREDO^1^ and Erica-tool^2^). A common parameter used in these models is the concentration factor (CF), which relates the concentration of an element in an organism to the concentration in the medium under equilibrium conditions.

Swedish nuclear facilities perform assessments of the radiation dose to the public using the software PREDO (PREdiction of DOses from normal releases of radionuclides to the environment^1^). In PREDO, a marine food web model is implemented, in which phytoplankton are at the base of the food chain. Thus, phytoplankton CF are expected to be important for estimates of internal radiation dose from radionuclides which reach humans through the food chain. The dependence of the estimated radiation dose on the phytoplankton CF for radioisotopes of the studied elements was studied in this work, to quantify this importance.

A previous screening using PREDO, based on actual releases from Swedish nuclear facilities to the marine environment^3^, resulted in a list of 15 radionuclides which are together responsible for more than 99% of the calculated radiation dose to humans. Literature studies of phytoplankton CF for the corresponding elements showed that most of them had already been subject to extensive studies. The determination of phytoplankton CF for two of the less well studied elements (Mn and Zn) is reported in previous work by the authors^4^. Furthermore, two isotopes of antimony (^124^Sb and ^125^Sb) were on the list. Calculations performed by the nuclear power plants for different realistic scenarios indicate that isotopes of ruthenium and nickel could also be among the important contributors to the dose to the public.

The literature was searched for experimentally derived data on the set of elements which were thus deemed to be of radioecological relevance, and these literature studies show that the phytoplankton CF for nickel, ruthenium and antimony are not well established.

Publications by the International Atomic Energy Agency (IAEA)^5–7^ and the Wildlife Transfer Database^8^ cover most of the literature on concentration factors. In addition to screening these documents, a literature search was performed using *Web of Science* (core collection) with the search phrase “[*element*] AND “phytoplankton” AND (“concentration factor” OR “concentration ratio” OR bioaccumulation OR bioconcentration)”. No further relevant publications or data was found for nickel, ruthenium or antimony, so it was concluded that the IAEA technical documents cover all the published literature on CF for the studied elements. The values recommended in the three latest relevant IAEA technical documents are listed in Table 1, along with references to the primary sources when available. The CF value for nickel in IAEA TECDOC 422^6^ is calculated from nickel concentrations from Refs.^9–11^, i.e., these references do not state CF data. It is noted that the values stated in the oldest relevant IAEA publication (IAEA TECDOC 211^5^) are generally identical, information on their primary source and experimental conditions is lacking, and no reference to whether fresh or dry weight of phytoplankton biomass was used in the calculations of these values is provided. The reference for the phytoplankton CF value for antimony in IAEA TECDOC 422^6^ is IAEA TECDOC 211^5^, i.e. also lacking information on primary source and statement on fresh/dry weight. No phytoplankton CF value is available for antimony in IAEA TECDOC 479^7^ and the values for nickel and ruthenium are very widely spread, with standard deviations exceeding the arithmetic mean value.

**Table 1:** Values of phytoplankton CFs for Ni, Ru and Sb from the relevant IAEA TECDOCs. All CFs are given in L/kg fresh weight.

	Mean	STD	N
Nickel			
IAEA TECDOC 211^5^	1000		
IAEA TECDOC 422^6^ (Primary sources:^9–11^)	3000		
IAEA TECDOC 479^7^ (Primary sources;^14, 15^)	570	740	3
Ruthenium			
IAEA TECDOC 211^5^	1000		
IAEA TECDOC 422^6^ (Primary source:^16^)	200,000		
IAEA TECDOC 479^7^ (Primary sources:^14, 17^)	6700	8500	3
Antimony			
IAEA TECDOC 211^5^	1000		
IAEA TECDOC 422^6^ (Cited source:^5^)	1000		

The arithmetic mean value, the number of samples (N) and the standard deviation (STD) and are listed if available. Primary sources are stated (if available).

In conclusion, there is very little data on phytoplankton CF available for the three elements nickel, ruthenium and antimony, while they are nevertheless relevant for realistic dose predictions. Hence, this work aims to complement the scarce data through experimental determination of these CF in a representative model organism. In addition, we aim to quantify any differences in the studied CF between the two main marine environments around the Swedish coasts, i.e. saline and brackish waters.

The diatom *Phaeodactylum tricornutum Bohlin (Bacillariophyceae)* was chosen as model organism for the study. Diatoms are by far the largest group of phytoplankton in the seas around Sweden^12^, and *P. tricornutum* is a useful representative of the group in this context since it is a cosmopolitan diatom that thrives equally well in saline and brackish waters^13^.

## Materials and methods

### Computational sensitivity studies

The aquatic model in the software PREDO^3^ was used to assess to which extent the committed dose from each of the investigated radionuclides is a result of exposure through the marine food chain. In PREDO, the marine ecosystem is represented by a box model including radionuclide transfer between different geographical regions, from the water column to marine biota and between different trophic levels within the food web (as described in, e.g.,^18^) ultimately leading to exposure of humans through intake of food of marine origin. Other marine exposure paths, such as swimming and inhalation of sea spray, are also included in the model. These exposure paths are independent of the food chain and thus independent of the CFs. The transformation of radionuclides to other nuclides through radioactive decay is also included in the model.

**Figure 1:**
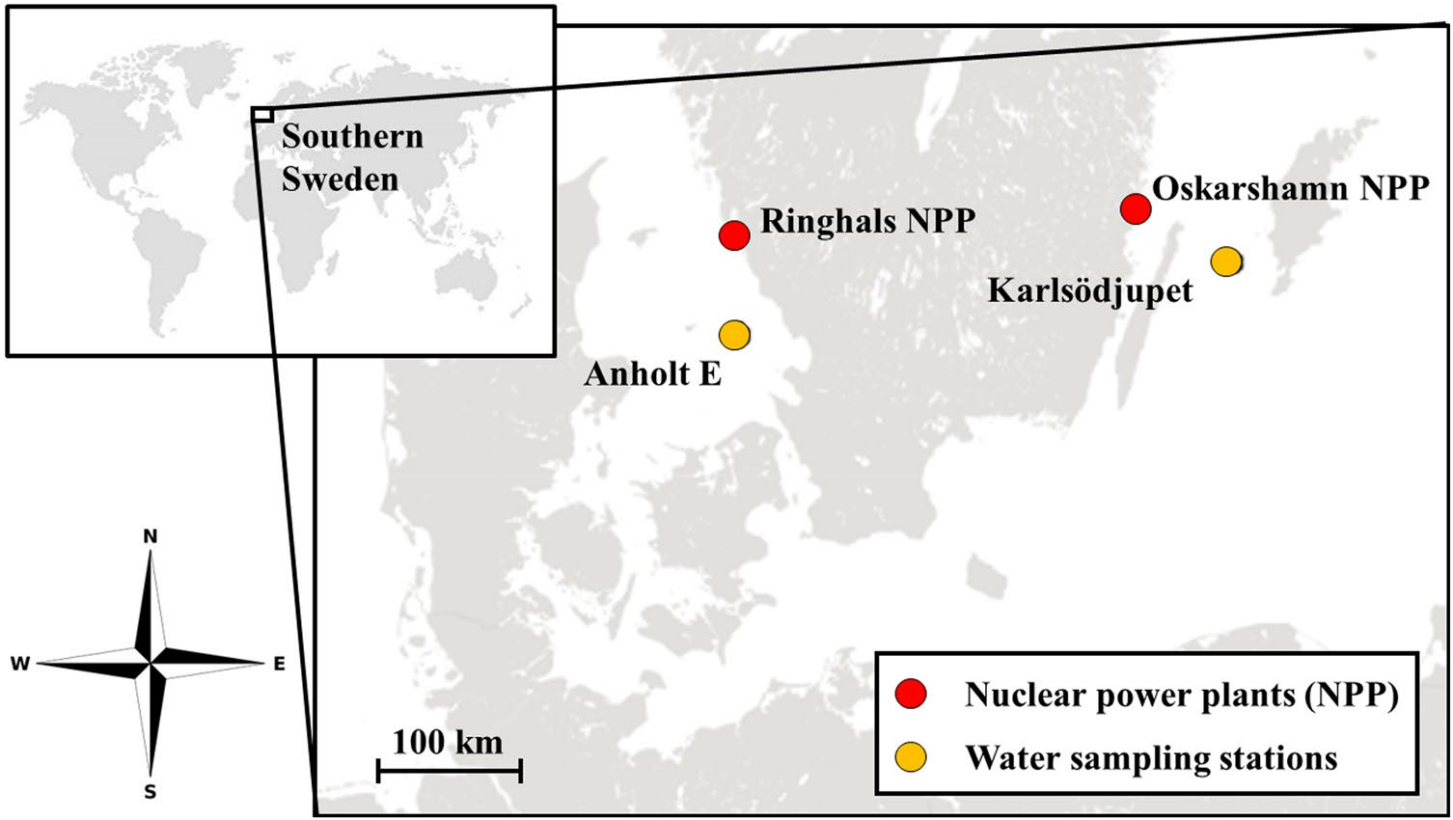
The location of the water sampling stations Anholt E (saline water, Kattegat) and Karlsödjupet (brackish water, Baltic Sea). The locations of the Ringhals and Oskarshamn nuclear power plants (NPP) are also indicated.

The PREDO model for the marine environment at the Ringhals nuclear power plant (NPP) located on the west coast of Sweden (see Fig. 1) was used. The annual dose after 100 years of release of 1 Bq/year is calculated and then normalized to actual releases. This dose was calculated for adults, children and infants of four different human population groups (fishing, hunting, farming and vegetarian families), according to the assumptions on habits and diet for the different families^19^. The implemented CF for each of the studied elements (Ni, Ru and Sb) was varied by a factor 10 and 1/10 respectively, to assess to which extent the dose is proportional to the CF, i.e. that exposure stems primarily from the marine food chain. The dose was only calculated for one nuclide of each of the elements (^63^Ni, ^106^Ru and ^125^Sb) since the same CF applies to all isotopes.

### Seawater sampling and analysis

Seawater samples were collected to be used as a growing medium for the experiment with a composition representative of the studied marine environments. The Swedish Meteorological and Hydrological Institute (SMHI) collected seawater samples in February 2022, at the regular sampling stations Anholt E (Kattegat, 56° 40.11 N, 12° 06.63ʹ E, sampling depth 50 m, water depth 63 m, salinity 33‰) and Karlsödjupet (Baltic sea—57° 07.03ʹ N, 17° 40.12ʹ E, sampling depth 80 m, water depth 110 m, salinity 10‰)^20^, see Fig. 1. The sampling stations in question were chosen because of their proximity to the Ringhals and Oskarshamn NPP, and because they represent the two main marine environments surrounding Sweden (saline and brackish water). The origin of the seawater used in the following experiments, i.e. whether the seawater comes from the Anholt E or Karlsödjupet sampling station, will henceforth be referred to as “water origin”.

The samples were collected using a water sampler (Hydro-Bios Free Flow Water Sampler) and immediately poured into 5L cans (Fisherbrand™ Polyethylene Narrow Neck Jerry Can) and refrigerated under dark conditions. Sampling was carried out according to methods recommended within the HELCOM COMBINE program^21^. The cans were previously unused and rinsed thrice with ultrapure (Milli-Q ®) water prior to use.

Natural background concentrations of Ni, Ru and Sb in the water samples used as growing medium were determined, primarily to assess to which extent the added element concentrations would disturb the natural concentrations. The concentrations were measured by ICP-MS (Thermo iCAP Q) using the standard addition method^22^. The values were corrected for instrumental background. Standards of 0, 1, 5 and 10 ppb of the respective elements were prepared from 10 ppm stock solutions (UltraScientific, CPA Chem, VGA Labs). Seawater samples were divided in four sub-samples that were diluted 1.7 times with 0.5 M HNO_3_ (Suprapur Merck) containing one standard for each sub-sample and internal standards of 2 ppb Sc, In and Bi. Mass intensity readings at masses 60, 104 and 121 were taken for Ni, Ru and Sb, respectively. Natural isotopic composition was assumed for the calculation of total element concentrations. The detection limit and accuracy of the method depend on concentration and the atomic weight of the element in question, and the accuracy (1σ) for each measurement is stated separately in the Results section.

Natural background concentrations of gamma-emitting radionuclides in the water samples was assessed by a 3-day measurement on 1.5 L of water from each water origin, using a detector system consisting of a high-purity germanium coaxial detector (Ortec GEM 50P4, Ametek, USA) with a relative efficiency of 52%, and a digital signal processor (DSPEC jr 2.0, Ametek, USA) providing an energy resolution of 1.65 keV at 1.33 MeV. Expected background concentrations of naturally occurring radioactive elements and ^137^Cs pollution remaining from the Chernobyl accident were detected, but no background radioactivity corresponding to the currently studied radionuclides. Natural background concentrations of beta-emitting radionuclides were assessed by a 10 min measurement on 5 mL of water from each water origin using a Liquid Scintillation Counter (Packard Tri-Carb® 2100TR, Perkin-Elmer, USA) with an energy range of 0–2000 keV, and 15 mL of liquid scintillation cocktail (Ultima Gold™, Perkin-Elmer, USA), mixed in a polyethylene scintillation vial (Selecta). No background concentration of any beta-emitting radionuclides was detected.

### *P. tricornutum* cultures

A non-axenic strain of *P. tricornutum* was procured from the algal bank at the University of Gothenburg (https://www.gu.se/en/marina-vetenskaper/about-us/algal-bank-gumacc). The bacteria were approximately one attached bacterial cell per 10 algal cells in a stationary culture (quantified by manual counting under microscope). No free-living bacteria were observed. The bacteria’s contribution to the total biomass was assumed to be negligible, due to their small size and that the measured algal cell dry weight was found to agree well with literature values. The *P. tricornutum* were cultured in nutrient-enriched, sterile filtered (not autoclaved) seawater samples, contained in 50 mL polystyrene cell culture flasks with vented caps (Thermo Scientific™ Nunc™ EasYFlask™, Nunclon™ Delta surface plasma treated). The culture flasks were previously unused and rinsed thrice with ultrapure (Milli-Q ®) water prior to use.

Sterile filtering was performed directly into the cell culture flasks, immediately before starting the cultures, using 0.2 μm polycarbonate membrane filters (Cytiva Whatman™ Cyclopore™). The nutrients (8.8‧10^–4^ M NaNO_3_, 3.6‧10^–5^ M NaH_2_PO_4_ and 1.1‧10^–4^ M Na_2_SiO_3_) were added to the sterile filtered seawater in the proportions of the f/2 medium^23^, however without vitamins or trace elements. Trace elements and vitamins were avoided in order to keep the water composition as representative as possible for the in-situ conditions in the marine environment. Preliminary experiments showed that excluding the trace elements and vitamins still yielded a satisfying growth rate of the *P. tricornutum*. Addition of nutrients was necessary to ensure exponential growth and is common practice in trace element metabolism experiments^4, 24–26^. The filtering funnels were washed with hot water and soap for several hours and thereafter rinsed thrice with distilled water before re-use; all other filtering equipment was single-use.

The radionuclides were added as solutions of NiCl_2_, RuCl_3_ and SbCl_3_ respectively. The Ni solution (PerkinElmer Life and Analytical Sciences, Boston, MA, USA) had an activity concentration of 60 MBq/L (± 10%) and a carrier concentration of 7.0 Ni_total_/^63^Ni. The RuCl_3_ and SbCl_3_ solutions (Eckert & Ziegler Strahlen- und Medizintechnik AG, Germany) both had an activity concentration of 500 GBq/L (± 15%) and inactive carrier concentrations of 790 Ru_total_/^106^Ru and 130,000 Sb_total_/^125^Sb respectively. The added radionuclide concentrations and the added stable carrier concentrations for the respective solutions are listed in Table 2, together with the results of the seawater analysis and the final total element concentrations, i.e. the sum of the added and measured element concentrations. The ruthenium and antimony solutions were strongly acidic (6 M HCl), so after addition to the seawater, NaOH solution was added to restore pH to 8 as it was before the radionuclide additions. The pH was monitored by the use of pH indicator sticks (Fisherbrand®, pH Indicator Paper Sticks, pHix 0–14) and remained constantly at 8 ± 0.5 throughout the experiment.

**Table 2:** Concentrations of Ni, Ru and Sb.

Element	Nickel	Ruthenium	Antimony
Radionuclide concentration [kBq/L]	36 ± 3.6	1000 ± 150	5.4 ± 0.8
Radionuclide concentration [pM]	280 ± 28	210 ± 30	0.42 ± 0.06
Added stable carrier concentration [nM]	2 ± 0.2	160 ± 20	53 ± 8
Measured element concentration at Anholt E [nM]	84 ± 5	43 ± 2	3.3 ± 0.8
Measured element concentration at Karlsödjupet [nM]	48 ± 7	13 ± 1	< 0.5*
Total element concentration (Anholt samples) [nM]	86 ± 5	200 ± 20	56 ± 8
Total element concentration (Karlsödjupet samples) [nM]	50 ± 7	170 ± 20	53 ± 8

First the added radionuclide and stable carrier concentrations (Note the different prefixes for radionuclide and stable carrier concentrations), then the measured concentrations in the seawater samples, measured using ICP–MS. Given uncertainties for the measurements are one standard deviation and given uncertainties for the radionuclide and stable carrier concentrations are those given by the radionuclide providers. The listed total element concentrations include the radionuclide, stable carrier and measured natural background concentrations.

*The concentration of antimony at the Karlsödjupet sampling station was below the detection limit of 0.5 nM.

After addition of nutrients and radionuclides and, where necessary, adjustment of pH, the culture growth was started by adding 1 mL of stock *P. tricornutum* culture to 29 mL of the solution. The resulting initial *P. tricornutum* cell concentration in the cultures was approximately 25‧10^3^ cells/mL. The stock solution composition and background element concentration in the initial phytoplankton population (approximately 1/40 to the final population) were deemed to be negligible and were thus not quantified. Three separate cultures were grown for each radionuclide and water origin (18 samples in total), for statistical purposes.

To provide a control for the experiment, identical control samples (also 18 in total) with the same water origins, nutrients and radionuclide additions, but with no *P. tricornutum,* were prepared. These control samples were used for assessing the amount of radioactivity sorbed onto the filters later used for separating the *P. tricornutum* from the medium.

The cultures and control samples were subjected to controlled light conditions; cold-white (6000 K) LED illumination was provided at an intensity of 200 µEm^-2^ s^−1^, similar to what has been used in previous experiments^4, 25, 27^ and (at the surface) slightly above optimal conditions for phytoplankton growth^28^. Illumination was provided 12 h/day, representative of the average daylight period. The temperature varied slightly, from 20 °C at the beginning of the light period to 25 °C at the end of the light period. These variations were caused by the illumination, but were deemed unimportant for the results, since the metabolism of *P. tricornutum* has been shown to be relatively insensitive to temperature fluctuations in this interval^13^. In preliminary experiments, constant gentle shaking was applied, which however affected neither the growth rate nor the metal uptake. In the reported experiments, the culture flasks were not shaken, except for a gentle swirling when the growth was assessed, at two-day intervals.

An Automated Cell Counter (Countess, ThermoFisher Scientific) was used for assessing *P. tricornutum* concentration and growth. A 100 µL aliquot was collected from each culture flask and fixated with 2 µL of acidic Lugol’s solution for *P. tricornutum* counting. This was performed at approximately two-day intervals, until a *P. tricornutum* concentration of approximately 10^6^ cells/mL was reached, generally within 6–8 days.

### Concentration factor determination

The entire 30 mL samples were filtered when the desired *P. tricornutum* concentration had been reached (i.e. 6–8 days after inoculation), and the radioactivity of the different fractions was measured, whereafter CFs were calculated using

1 $$CF= \frac{{{A}_{plankton}-A}_{control}}{{m}_{plankton}}{\left(\frac{{A}_{medium}}{{V}_{medium}}\right)}^{-1},$$

where *V*_*medium*_ and* A*_*medium*_ are the volume and the activity of the filtrate (the growing medium) respectively,* A*_*plankton*_ is the activity of the filters with *P. tricornutum*, *A*_*control*_ is the activity on the filters used for the control samples and *m*_*plankton*_ is the total *P. tricornutum* dry mass.

### Dry weight determination

The dry weight of the *P. tricornutum* was determined by filtering 25 separate *P. tricornutum* cultures (13 with Anholt water and 12 with Karlsödjupet water) with known volume and cell concentration through polycarbonate membrane filters (Cytiva Whatman™ Cyclopore™, pore size 1 µm, diameter 25 mm) which had been pre-soaked in NH_4_HCO_2_ (Ammonium formate, Acros Organics, 99%) solution (isotonic with the sea water), dried and weighed.

After *P. tricornutum* culture filtration with a transmembrane pressure of 17 kPa, the filters and *P. tricornutum* were rinsed twice with 5 mL of the isotonic NH_4_HCO_2_ solution. Rinsing was performed to avoid adding salt mass to the filters, NH_4_HCO_2_ solution was used since it evaporates completely at 60 °C, and the solution was prepared to be isotonic with the sea water to avoid osmotic rupture or shrinking of the rinsed plankton. After rinsing, the filters were dried overnight at 60 °C and immediately weighed again. The dry weight of a single *P. tricornutum* cell was calculated by dividing the filter mass gain by the number of cells^25^.

For the concentration factor determination, the total *P. tricornutum* dry mass in each of the experimental samples was calculated as the product of the dry weight per cell and the number of cells in the respective sample as assessed by automated cell counting.

### Radioactivity measurements

For the activity measurements, the samples with added radionuclides (both *P. tricornutum* samples and control samples) were filtered through polycarbonate membrane filters (Cytiva Whatman™ Cyclopore™, pore size 1 µm, diameter 25 mm) with a transmembrane pressure of 17 kPa. The filters were then rinsed twice with 5 mL of sterile filtered seawater before activity measurements.

Great care was taken to prevent dust particles from falling into the flasks, onto which the studied elements might possibly adsorb. Such particle contamination would lead to a disproportionately high measured radioactivity in the fraction caught on the filters. However, the risk could not be entirely eliminated.

The activity measurements on the filters and the filtrates were performed differently for the different radionuclides, due to their different decay characteristics.

The activity of ^125^Sb could be assessed directly by gamma counting, using a detector system consisting of a high-purity germanium coaxial detector (Ortec GEM 50P4, Ametek, USA) with a relative efficiency of 52%, and a digital signal processor (DSPEC jr 2.0, Ametek, USA) providing an energy resolution of 1.65 keV at 1.33 MeV. The counting time was chosen to ensure a counting uncertainty less than 3% (1σ). Geometric efficiency corrections were made using the EFFTRAN v. 4.2 computer code^29^ and previous measurements of a calibration source.

^106^Ru (t_1/2_ = 371.5 d) decays by beta emission only to ^106^Rh (t_1/2_ = 30.1 s), which in turn decays by beta emission to stable ^106^Pd. The latter decay is accompanied by gamma emissions, which enables the indirect detection of ^106^Ru decay through gamma spectrometry^30^. The relatively short half-life of ^106^Rh means that a secular equilibrium is established already within minutes, i.e. the decay rate of ^106^Ru and ^106^Rh are then identical. Thus, the activity of ^106^Rh was measured using the same detector system as for ^125^Sb and the counting time was chosen to ensure a counting uncertainty less than 3% (1σ).

The activity adsorbed in the culture flasks was measured for 25 flasks, by placing each culture flask on the detector and, for interpreting the results, making suitable geometry corrections using EFFTRAN v. 4.2^29^. Also for the flasks, the counting time was chosen to ensure a counting uncertainty less than 3% (1σ).

^63^Ni is also a beta emitter only, which decays directly to stable ^63^Cu with no accompanying gamma emissions. Hence, the activity of ^63^Ni in the water and *P. tricornutum* fractions were measured by liquid scintillation counting using a Liquid Scintillation Counter (Packard Tri-Carb® 2100TR, Perkin-Elmer, USA) with an energy range of 0–2000 keV, a liquid scintillation cocktail (Ultima Gold™, Perkin-Elmer, USA) and polyethylene scintillation vials (Selecta). For water samples, 5 mL of the water was mixed with 15 mL of the scintillation cocktail and for measurement of activity on filters with *P. tricornutum* and control filters, the entire filter was immersed in 20 mL scintillation cocktail and shaken well for 30 s. Visual inspection confirmed that all but a negligible fraction of the *P. tricornutum* was dispersed in the scintillation cocktail. Quenching corrections were performed by adding a known amount of activity to a separate set of vials containing similar water/cocktail, filter/cocktail and *P. tricornutum* /filter/cocktail mixtures and measuring the corresponding count rates. Direct proportionality of the count rate to the activity was confirmed by count rate measurements for a range of known activities, spanning the range of measured activities in the different samples. Counting was carried out for 2 min for each sample, and the count rate at energies below 67 keV were used for the activity calculations. Six repeated measurements on the same sample showed an uncertainty of 1.4% (1σ).

## Results and discussion

### Computational sensitivity studies

**Figure 2:**
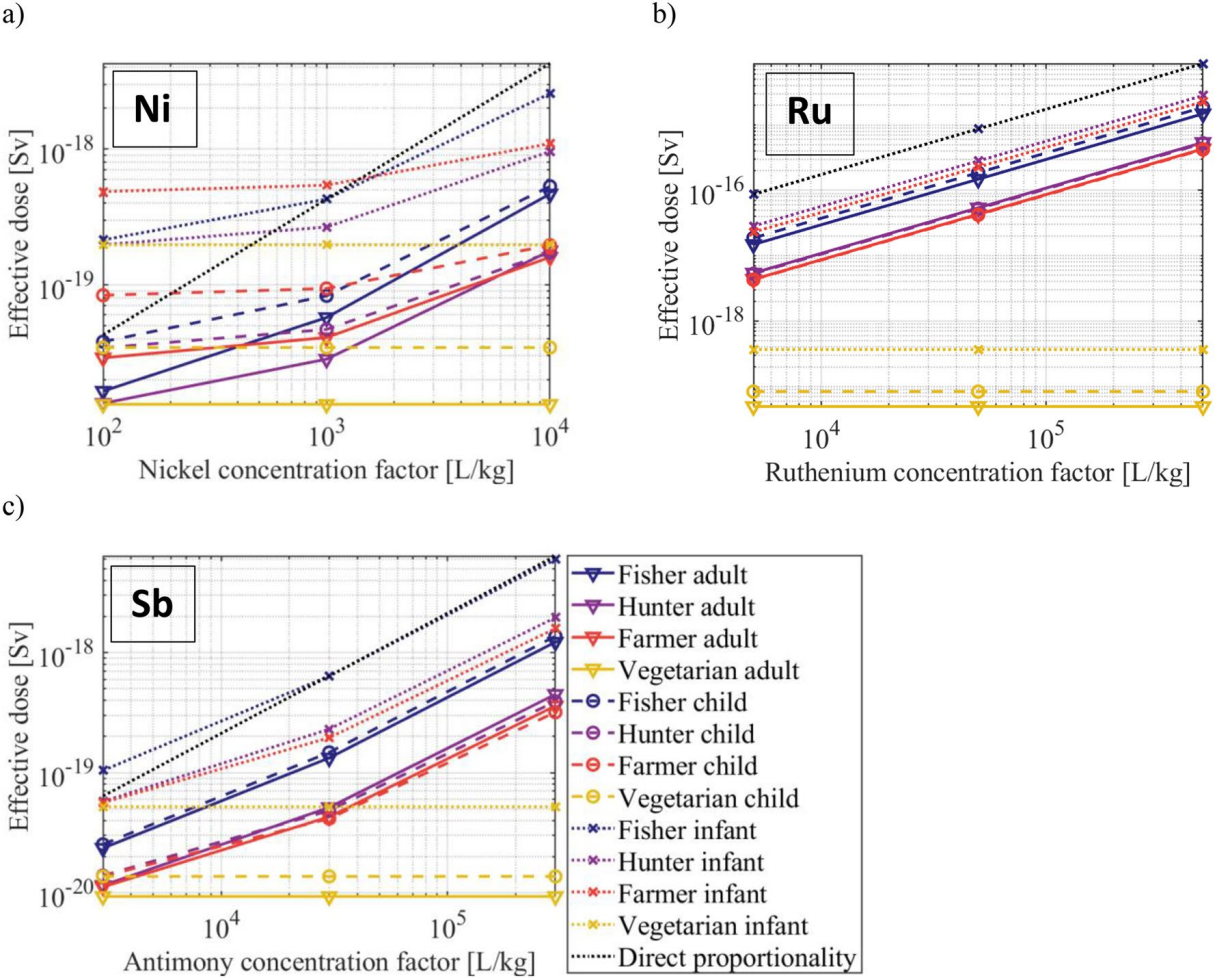
Effective dose committed to each of the representative families listed in the plot legends (fishing, hunting, farming and vegetarian) and each age category (adult ▼, child ● and infant ×), calculated for (**a**) ^63^Ni, (**b**) ^106^Ru and (**c**) ^125^Sb. In each plot, a black dashed line representing direct proportionality has been added for reference.

The effective doses received during 1 year for three age groups and four population groups were calculated using PREDO for ^63^Ni (Fig. 2a), ^106^Ru (Fig. 2b) and ^125^Sb (Fig. 2c). The definitions of the age categories and population groups are given in reference^19^. As previously stated, the values are calculated assuming a hypothetical continuous release of 1 Bq/year for 100 years and are thus only intended for intercomparison. For all studied population groups and elements, the calculated dose was higher for lower age, which is expected due to the age dependence of the used dose coefficients^1^ caused by differences in biokinetics between different age groups^31^. In general, the fishing family received the highest dose, for most studied age categories and elements, with some exceptions for nickel. For ruthenium, the calculated dose is significantly lower for the vegetarian family compared with the other families, and the dose for the other families is also almost directly proportional to the ruthenium CF, indicating that the dose from radioisotopes of ruthenium arises almost exclusively by ingestion. For antimony, the difference between the vegetarian and other families is less pronounced, whereas for nickel, the CF is less important for the calculated dose, indicating more exposure through other paths than the marine food chain. At the currently implemented value of the nickel CF, 1000 L/kg, the dose received by the farmer child is almost 3 times higher than that of the vegetarian child, so the ingestion exposure pathway is still dominant, indicating that for nickel, as well as for ruthenium and antimony, the CF is essential for calculation of radiation dose to all non-vegetarian population groups.

### Element concentrations in seawater samples

Results of the ICP-MS analysis of the concentrations of nickel, ruthenium and antimony in the seawater samples are listed in Table 2.

The measured nickel concentration is almost an order of magnitude larger than concentrations previously found in Kattegat and the Baltic Sea (5–14 nM)^32^, which could be due to contamination during sampling or storage^33^, or to increased pollution since these values were obtained in 1982. Seawater samples retrieved in February 2021 by the same sampling methodology, in the same locations (which were however not used in this experiment) showed even higher concentrations (134 nM). Even if the measured concentrations are due to sample contamination, and the true Ni concentrations are as low as reported in 1982^32^, the added Ni concentration (2 nM) does not alter the total Ni concentration in the seawater samples to a large extent, so the CF values obtained in the reported experiments are likely to be representative for the in-situ conditions in the studied marine environments.

Data on ruthenium concentrations in Kattegat or the Baltic Sea could not be found. The ruthenium concentration in heavily trafficked waters in the Mediterranean Sea has been measured to be 0.5 nM^34^, which is much lower than our measured values. However, ruthenium pollution occurs to an important extent through wear of ruthenium containing alloys which are frequently used in high-wear applications^34^, resulting in small ruthenium-containing particles which may have been included in our analysis, but not included in^34^ due to finer filtering. Other possibilities include contamination of the samples or an actual much higher ruthenium pollution in the studied marine environments. Irrespective of the actual value, the addition of 160 nM ruthenium in the reported experiments results in a significant increase of the ruthenium concentration in the used growing medium. If ruthenium uptake in *P. tricornutum* is concentration dependent or even reaches a saturation level, the obtained CF values are thus conservative. The ruthenium content was not assessed in the samples taken in February 2021.

The antimony concentration in the Baltic Sea has been measured to be 0.3–0.8 nM^35^ and 1.7 nM in the North Atlantic^36^ which is similar to our measured data, and to the antimony concentration found in the water samples retrieved in February 2021 (0.7 nM). Similarly to the ruthenium case, the concentration of antimony added as radionuclide and stable carrier results in a significant increase of the total concentration above the background, so that the obtained CF values are conservative if the CF would be concentration dependent.

### *P. tricornutum* dry weight

The dry weight of one *P. tricornutum* cell was found to be 23 ± 5 pg (1σ). No statistically significant difference was found between the dry weight of *P. tricornutum* grown in water from Anholt E or Karlsödjupet respectively, so the same value was used in the concentration factor calculation for water from both origins. The found value agrees well with previously published values; 22.6 pg per cell^26^ and 23 pg organic mass per cell^37^.

### Concentration factors

A CF was calculated individually for each sample according to Eq. (1), where *m*_*plankton*_ was calculated as the product of the plankton cell concentration, the culture volume and the *P. tricornutum* cell dry weight. The final *P. tricornutum* cell concentration at the time of filtering was 1.1‧10^6^ ± 0.4‧10^6^ cells/mL. For each element and water origin, a mean value and standard deviation was calculated from the three CF thus obtained. These values are listed in Table 3. For nickel, the mean activity measured on the control filters was less than 1% of that caught on filters with *P. tricornutum*. For ruthenium and antimony, the corresponding number was 25% and 21% respectively. Given that the *P. tricornutum* were in a state of exponential growth, the obtained values are conservative estimates of the CF.

**Table 3:** Concentration factors (CF) in *P. tricornutum* for nickel, ruthenium and antimony.

Element	Water origin	CF (dw) [L/kg]		CF (fw) [L/kg]		RSD (%)
Nickel	Anholt E	6400 ± 1900		1200 ± 300		30
	Karlsödjupet	6100 ± 800		1100 ± 140		13
Ruthenium	Anholt E	15,000 ± 11,000		2700 ± 2000		75
	Karlsödjupet	20,000 ± 8000		3600 ± 1400		40
Antimony	Anholt E	190 ± 150		34 ± 27		81
	Karlsödjupet	890*		160*		

CFs and uncertainties (one standard deviation) are given in terms of dry weight (dw). Relative standard deviations (RSD) are given in %. CFs are also given in L/kg fresh weight (fw) for comparison with literature values below, where the conversion factor of 0.18 dry weight to fresh weight recommended by IAEA^[7]^ has been used.

*No standard deviation is given because one of the triplicate samples was excluded.

For the gamma emitting nuclides, ^106^Ru and ^125^Sb, the activity remaining in the culture flasks was ≤ 3.3% of the total recovered activity, except for the samples with *P. tricornutum* growing in water from Karlsödjupet with an addition of ^106^Ru, in which case the activity remaining in the culture flask was, on average, 15%. The recovered radioactivity (i.e. the radioactivity detected in the medium, *P. tricornutum* and flasks) was equal to the added radioactivity within the uncertainty of the radioactivity concentration of the stock solution, which was 15%, as given by the radionuclide provider. Due to the difficulty of measuring the activity of the culture flasks with the scintillation detector, which would have demanded the flask to be cut in small pieces and introduced in a scintillation vial, the activity remaining in the flasks where *P. tricornutum* had been cultured with an addition of ^63^Ni was not measured. The average recovered activity of ^63^Ni in the medium and *P. tricornutum* fractions was 91%. Furthermore, since the measured radioactivity in the *P. tricornutum* filters, the control filters and the medium were used relatively to each other, absolute radioactivity quantities are not important for the CF determination. Although sorption on the walls occurs, this does not affect the CF determination. Due to the dynamic equilibria in the sorption process, the distribution of radionuclides between the *P. tricornutum* and the surrounding medium will be unaffected by sorption on the walls.

#### Nickel

The difference between the CFs calculated for the water samples from Anholt E and Karlsödjupet respectively is not statistically significant (the difference is smaller than one standard deviation), which suggests that the nickel CF is not concentration dependent within the relevant nickel concentration range, since the Ni concentration at Anholt E is approximately twice that at Karlsödjupet. Both values (1200 L/kg and 1100 L/kg fresh weight, respectively) are also, within one standard deviation, identical to the geometric mean of the values listed by IAEA^7^ (570 ± 740 L/kg fresh weight).

#### Ruthenium

The calculated CFs for ruthenium are also, within one standard deviation, identical for the two water sampling sites. The total ruthenium concentration differed by 18% between the media based on Anholt E and Karlsödjupet water respectively. The absence of a statistically significant difference thus suggests that the CF is at least not strongly concentration dependent, and there are no signs of saturation effects, at the comparatively high ruthenium concentrations present in the experiment. Since the addition of ruthenium was large compared with the natural background concentrations, this conclusion may not hold for the lower ruthenium concentrations found in the studied marine environments, but it is unlikely that there would be a concentration dependence at low concentrations but not at higher. The values, 2700 L/kg and 3600 L/kg fresh weight, for Anholt E and Karlsödjupet respectively, are both within one standard deviation from the mean value given by IAEA TECDOC 479^7^ (6700 ± 8500 L/kg fresh weight). It should be mentioned that the very high value recommended in IAEA TECDOC 422^6^ (200,000 L/kg fresh weight), which is currently implemented in PREDO, refers to^16^ who in turn refer to a conference paper from 1965^38^ where it is explicitly mentioned that the data, established for Caribbean ecosystems, are preliminary. Thus, our results are very likely to represent a better estimate of the typical phytoplankton CF in Swedish marine ecosystems.

#### Antimony

Finally, the CF values for antimony calculated from our measurement data are quite widely spread. For one of the samples with water from Karlsödjupet, the measured activity in the *P. tricornutum* fraction was an order of magnitude larger than for the other two samples, although the *P. tricornutum* concentration was similar. We assume that this was due to some foreign particle(s) entering the culture, onto which much antimony was adsorbed. This sample was therefore excluded from the analysis. The mean value for Anholt E (34 L/kg fresh weight) has an 81% RSD and the mean value for Karlsödjupet (160 L/kg fresh weight) is more than two standard deviations larger, however, given that no standard deviation could be calculated for the Karlsödjupet value, it is not possible to determine if the difference is statistically significant. No value is given in IAEA TECDOC 479^7^, while the recommended value in IAEA TECDOC 422^6^ (1000 L/kg) is the same as in IAEA TECDOC 211^5^, for which no original source is stated. Furthermore, no information is given on how the value was obtained or whether it refers to phytoplankton fresh or dry weight. Thus, our values probably represent the first published CF measurement data for antimony in phytoplankton.

## Conclusions

The results from the computational sensitivity study with the marine ecosystem model in PREDO showed that correct CFs for phytoplankton are important for a correct dose assessment for the different population groups, except for vegetarians for which other sources than seafood ingestion dominated the dose.

Conservative estimates of the fresh weight CFs in the marine diatom species *P. tricornutum* are 6400 ± 1900 L/kg dry weight for nickel, 20,000 ± 8000 L/kg dry weight for ruthenium and 890 L/kg dry weight for antimony (where no standard deviation could be calculated). Differences between the CF between waters of different origin are, to the precision of our measurements, not significant, indicating that the same CF can be used for all dose estimates concerning Swedish marine environments.

The estimates for nickel and antimony do not deviate strongly from the values of these CF which have been published previously and implemented in PREDO, so this work serves to reinforce the scientific basis of the phytoplankton CF used for assessments of dose to the Swedish public from radioactive isotopes of nickel and antimony. The phytoplankton CF estimate for ruthenium is similar to the value given in the latest relevant IAEA report on CF^7^, but significantly lower than that currently implemented in PREDO, so this work reinforces the scientific basis for abandoning the currently implemented ruthenium phytoplankton CF of 200,000 L/kg fresh weight for a lower value, which should ultimately result in more accurate estimates of radiation dose to the Swedish public from radioactive isotopes of ruthenium.

## Data Availability

Data is available upon reasonable request to the corresponding author.
